# Expanding the Applicability of Electroactive Polymers for Tissue Engineering Through Surface Biofunctionalization

**DOI:** 10.3390/biomimetics10020126

**Published:** 2025-02-19

**Authors:** Beatriz Leiva, Igor Irastorza, Andrea Moneo, Gaskon Ibarretxe, Unai Silvan, Senentxu Lanceros-Méndez

**Affiliations:** 1 Basque Centre for Materials, Applications and Nanostructures (BCMaterials), UPV/EHU Science Park, 48940 Leioa, Spain; beatriz.leiva@bcmaterials.net (B.L.); igor.irastorza@bcmaterials.net (I.I.);; 2 Physics Centre of Minho and Porto Universities (CF-UM-UP) and LaPMET—Laboratory of Physics for Materials and Emergent Technologies, University of Minho, 4710-057 Braga, Portugal; 3 Cell Biology and Histology Department, Faculty of Medicine, University of the Basque Country (UPV/EHU), 48940 Leioa, Spain; 4 Ikerbasque, Basque Foundation for Science, 48009 Bilbao, Spain

**Keywords:** PVDF, electroactivity, collagen, biofunctionalization, piezoelectricity

## Abstract

Polyvinylidene fluoride (PVDF) is a synthetic semicrystalline fluoropolymer with great potential for tissue engineering applications. In addition to its excellent mechanical strength, thermal stability, biocompatibility and simple processability into different morphologies, the relevance of PVDF-based materials for tissue engineering applications comes for its electroactive properties, which include piezo-, pyro- and ferroelectricity. Nevertheless, its synthetic nature and inherent hydrophobicity strongly limit the applicability of this polymer for certain purposes, particularly those involving cell attachment. In addition, the variable adhesion of cells and proteins to PVDF surfaces with different net surface charge makes it difficult to accurately compare the biological response in each case. In this work, we describe a method for the surface functionalization of PVDF films with biological molecules. After an initial chemical modification, and, independently of its polarization state, the PVDF films covalently bind equivalent amounts of cell-binding proteins. In addition, the materials retain their properties, including piezoelectric activity, representing a very promising method for the functionalization of PVDF-based tissue engineering approaches.

## 1. Introduction

Piezoelectricity refers to the ability of some materials to generate an electric potential in response to an applied mechanical stress. This phenomenon is the result of the internal structure of the material, which lacks a centre of symmetry. The effect is reversible, meaning that, when an electric field is applied to a piezoelectric material, a mechanical deformation occurs. In the year 1964, Fukada and Iwao reported the piezoelectric response of bone [1], and, since then, a number of body tissues, including tendon, vascular endothelium and skin, have been found to exhibit this property. The wide distribution of piezoelectricity among tissues of different origins suggests the biological importance of the coupling between mechanical deformation and electrical polarization. In fact, the beneficial impact of this type of stimulation on regeneration and tissue homeostasis have been widely reported over the last years [2,3,4,5,6,7], remaining however the precise mechanisms involved in the transduction of these signals into the cells and the signalling pathways involved in the biological response poorly described. In this context, the use of piezoelectric scaffolds shows great potential to faithfully biomimic the native environment and to promote an optimal integration of implanted materials into the host tissues.

Among synthetic piezoelectric materials, polyvinylidene fluoride (PVDF) is a semi-crystalline thermoplastic fluoropolymer that shows good biocompatibility, excellent mechanical properties, chemical stability, and easy processability into a number of different structures [8]. Depending on the preparation conditions used, PVDF can crystallize in five different forms (namely, α, β, γ, δ and ɛ), each one related to a different chain conformation [8]. Among these forms, the β-phase is the one that displays the highest dipolar moment and piezoelectric coefficients d_31_ (polarization in z axis per unit stress applied in x axis) and d_33_ (polarization in direction z axis per unit stress applied in z axis) and is therefore the most interesting for tissue engineering applications [9,10]. In addition, the exposure of the PVDF films to a high electric field through an electrically charged needle and a grid electrode, a process named ‘poling’, causes the alignment of the individual dipole moments of the molecules and results in films with a net positive and a net negative side [9]. Thereby, the versatility of PVDF makes it possible to produce scaffolds that accurately mimic the electroactive properties of native tissue. This renders the polymer a highly attractive choice for the fabrication of biomedical implants for tissue reconstruction, particularly bone. However, the synthetic composition and inherent hydrophobicity of PVDF raise considerable challenges that significantly restrict its use in biological contexts. In addition, the distinct electrostatic interaction of proteins with the surface of PVDF with different surface potentials is very likely behind some of the reported differential response of cells to them, such as the higher proliferation rate of certain cell types on positively and, specially, negatively poled PVDF films than on non-poled ones [11] or the impact of PVDF poling on the spreading of adipose mesenchymal stem cells [12]. In fact, slower adsorption and conformational differences in collagen deposited on non-poled PVDF films, compared with positively and negatively poled ones has been recently reported [13].

To exploit the full potential of PVDF in biomedical applications and, specifically, for the creation of biomimetic piezoelectric implants with improved cellular affinity, it is essential to overcome these limitations. To achieve this, a number of procedures for the covalent functionalization of PVDF have been reported over the last years. These protocols generally use an initial physicochemical activation step to transitorily decrease the inherent hydrophobicity of the polymer surface [14], followed by the grafting of crosslinkers, such as glutaraldehyde [15], 2-hydroxyethyl methacrylate (HEMA) [16], or acrylic acid [17,18,19]. This approach has been used for the immobilization of a number of biomolecules, including bovine serum albumin (BSA), protein A [20,21], collagen and vascular endothelial growth factor (VEGF), among others [20,21,22,23,24,25]. However, previous work in the field has either employed non-electroactive crystalline forms of PVDF or has completely neglected the electroactive properties of the material. With this in mind, in the present work, we describe the use of two different acrylic acids, namely, methacrylic (or MAA) and acrylic acid (or AAC), to functionalize the surface of β-phase PVDF films with different polarities (including non-poled with an average zero surface charge, positive and negative poled with either positive or negative average surface charge). We then activated the exposed carboxylic groups of the acids using a carbodiimide/N-hydroxysuccinimide (EDC/NHS) reaction for the covalent binding collagen type I to the surface. We subsequently performed a biophysical and chemical characterization of the functionalized PVDF surfaces along cell culture experiments using the functionalized PVDF substrates and primary human dental pulp stem cells (hDPSCs), a multipotent cell type isolated from wisdom teeth [5,26] to determine their biocompatibility.

The described immobilization of collagen on PVDF films results in a composite that mimics bone in the sense that it reproduces the electrical behaviour of native tissue through the piezoelectric response of PVDF while providing a biochemically relevant interface that enhances cell adhesion. Thus, our protocol expands the potential of this polymer for biomedical research applications, enhancing its functional versatility and biocompatibility, thus paving the way for its use in advanced tissue engineering strategies. Furthermore, since the developed method renders similar collagen densities independently of the surface potential of the PVDF film, while retaining the piezoelectric nature of the material, it will help us understand the transduction pathways involved in the response of cells to these signals.

## 2. Materials and Methods

### 2.1. Chemical Functionalization of the PVDF Membranes

**Figure 1:**
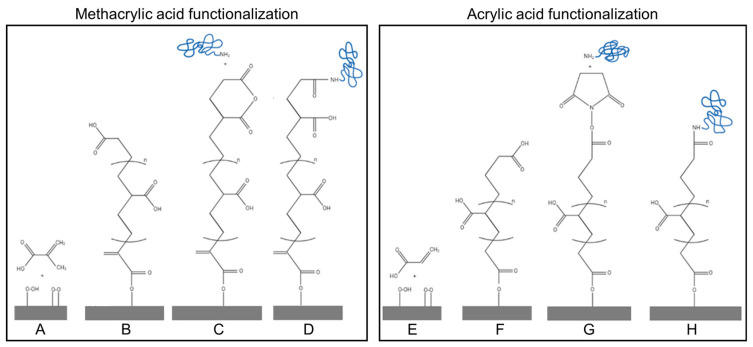
Schematic representation of the functionalization strategy: (**A**) Incubation of the activated PVDF surface with MAA solution. (**B**) PMAA polymerization by ester bond formation. (**C**) Acid activation with EDC/NHS. (**D**) Amide bond formation with the protein of choice. (**E**) Incubation of the activated PVDF surface with AAC solution. (**F**) PAAC polymerization by ester bond formation. (**G**) Membrane activation by EDC/NHS method. (**H**) Amide bond formation with the protein of choice.

For the chemical functionalization of the PVDF surfaces, a modified graft polymerization method based on the protocol developed by Kaur et al. [27] was followed. The method is based on the addition of hydroxyl groups (-OH), followed by the reaction of either methacrylic (MAA) or acrylic (AAC) acid molecules by a polymerization reaction. The carboxylic groups of the acids are subsequently activated by the EDC/NHS method and react with amine groups present in the proteins, collagen type I in our experiments [28] (Figure 1). Firstly, PVDF membranes (purchased from PolyK State College PA, crystallized in β-phase and with a thickness of 0.1 mm and d_31_ > 25 pC/N, d_33_ > 20 pC/N) with different polarities, including neutral (PVDF (non-poled)), positively poled (PVDF (poled+)) and negatively poled (PVDF (poled−)) (Table 1), were cut into 15 mm diameter discs. The samples were then washed twice with deionized water and once with absolute ethanol, followed by air drying using a gentle flow of air to remove any remaining liquid. Next, the PVDF membranes were placed in a UV ozone cleaner (PSD-UV, Benchtop UV Ozone Cleaner, Novascan, Milwaukee, WI, USA), ensuring that the relevant surface (positive or negative side) of the poled films was facing upwards. Then, an oxygen atmosphere was applied during 30 s before illuminating the surfaces with UV light for 15 min in order to activate them. After that, the membranes were left exposed to air for 5 min to increase the formation of peroxides and hydroperoxide species before functionalization [29] and then immediately incubated for 1 h in different solutions (500–700 µL per cm^2^): 10% (*v*/*v*) methacrylic acid (MAA) for PVDF (poled+ and poled-), 8% (*v/v*) methacrylic acid for PVDF (non-poled), or 10% (*v/v*) acrylic acid (AAC) for poled and non-poled PVDF (Supplementary Figure S1). Before incubation, the MAA and AAC solutions were heated to 80 °C as described in Kaur et al., 2007 and Liang et al., 2013 [27,29] and then placed in a vacuum chamber for 5 min. Next, the solutions were exposed to a nitrogen flow for another 5 min to remove any remaining dissolved gas. After the incubation, the PVDF membranes with immobilized poly-methacrylic acid (PVDF-PMAA) or poly-acrylic acid (PVDF-PAAC) were washed with deionized water and soaked in a 0.1 M sodium hydroxide (NaOH) solution overnight with shaking in order to remove unreacted monomers and homopolymers. Finally, the functionalized membranes were washed with deionized water, air dried, and stored at room temperature until further use. Figure 1 depicts a schematic representation of the functionalization method.

### 2.2. Protein Immobilization on the PVDF-PMAA and PVDF-PAAC Membranes

To promote the adhesion and spreading of cells and to better mimic the native biological environment, we then immobilized type I collagen on the PVDF surfaces. To this end, the free carboxylic groups of the PMAA and PAAC molecules that were covalently bound to the PVDF films were activated by the carbodiimide/N-hydroxysuccinimide (EDC/NHS) method [29]. Briefly, the membranes were incubated in a solution containing 2 mM of 1-ethyl-3-(-3-dimethylaminopropyl) carbodiimide hydrochloride (EDC, 22980, Thermo Scientific, Waltham, MA, USA), 5 mM of N′, N′-dicyclohexyl carbodiimide (NHS, 24500, Thermo Scientific) and 10 mM (2-(N-morpholino) ethanesulfonic acid) (MES, M1317, Sigma, Burlington, VT, USA) at pH 4.5 for 15 min at room temperature and an approximate volume of 500 µL per cm^2^. Then, they were washed with deionized water and immediately incubated with a 300 µg/mL collagen type I solution (A1048301, Gibco, Carlsbad, CA, USA) for 24 h. To ensure homogeneous surface contact with the collagen solution, this incubation step was carried out with the films floating on a 500 µL drop of the solution with the PAAC- or PMAA-functionalized surfaces facing down. The biofunctionalized membranes were finally carefully washed twice with deionized water and stored for further experiments at 4 °C.

### 2.3. Physicochemical Characterization of the Membranes

We then performed the characterization of the materials in order to confirm the immobilization of the collagen type I molecules, obtained information on the properties of the resulting material, and determined its suitability for use in biological contexts.

Firstly, to gather information on the chemical modification of the surfaces and to ensure the presence of PMAA and PAAC molecules, the absorbance of the modified PVDF films was measured using a Cary 60 UV-Vis spectrophotometer (Agilent Technologies, Santa Clara, CA, USA). Briefly, circular fragments of the functionalized PVDF, each with a diameter of 15 mm, were placed in the light path of the instrument to collect absorption data, ensuring an identical measured area for all samples. Spectra of the samples were recorded in the 190 to 250 nm wavelength range, and the absorbance at 207 nm and 193 nm was used to determine the presence of PMAA and PAAC, respectively.

In turn, contact angle measurements were performed using an Ossila Contact Angle Goniometer (Brighton Science, Cincinnati, OH, USA). Here, a 5 µL droplet of distilled water was carefully deposited in the central region of the PVDF film. The droplet was then allowed to stabilize on the membrane surface for 5 s in order to ensure consistent spreading and equilibrium before capturing an image for analysis. The contact angle of each droplet was then estimated using the ImageJ software (version 1.54) and the Contact Angle plugin [31].

To quantify the density of collagen type I immobilized on the different PVDF films, we used a bicinchoninic acid (BCA) assay following the manufacturer’s instructions (71285-M Millipore, Burlington, MA, USA). Briefly, the PVDF membranes were incubated in a mixture containing bicinchoninic acid and copper (II) sulphate (CuSO_4_) in PBS during 30 min at 37 °C and 100% humidity. The measured signal comes from the colour change that occurs when Cu⁺ ions react with bicinchoninic acid and give rise to a purple complex. Accordingly, the colour intensity is directly related to the amount of protein present. We measured the absorbance of the samples using a plate reader (Infinite MNano+, Tecan, Männedorf, Switzerland) set at 562 nm and extrapolated the readout to a calibration curve made with native collagen type I (shown in Supplementary Figure S2).

The piezoelectric coefficient (d_33_) of both bare and functionalized PVDF films was measured using a d_33_ piezoelectric tester (YE2730A, Sinocera, Shanghai, China). These measurements were performed under controlled conditions at a frequency of 110 Hz and with an applied force of 0.25 N.

We further estimated the β-phase content after the chemical modification using Attenuated total reflectance Fourier transform infrared spectroscopy (FTIR-ATR). Spectra were collected over a wavelength range from 600 to 4000 cm^−1^ with a resolution of 4 cm^−1^, and a total of 64 scans were averaged to generate the final spectra. Bands at 766 cm^−1^ and 840 cm^−1^ were used to calculate the relative of the β-phase fraction [32].

In turn, the topographic characteristics of the PVDF surfaces and immobilized collagen fibres were analyzed using Scanning Electron Microscopy (SEM). For the preparation of the specimens, bare and collagen-coated PVDF films were chemically fixed using a 2% (*w/v*) glutaraldehyde solution for 10 min to preserve structural integrity. Subsequently, samples were subjected to a dehydration process using a graded ethanol series (50%, 75% and 100%), lasting 5 min at each step, after which the samples were left to air dry. Prior to imaging, samples were sputter-coated for 2 min, resulting in the deposition of a gold layer of approximately 15 nm. Finally, SEM images were acquired using a S4800, Hitachi High Technologies system at 10 kV and post-processed using ImageJ software [31].

### 2.4. Human Dental Pulp Stem Cell (Hdpsc) Isolation and Culture

Human dental pulp stem cells (hDPSCs) were isolated from the wisdom teeth of young healthy patients (ages between 18 and 30 years) following a previously published protocol [33]. Briefly, human teeth were collected and sterilized with 70% ethanol. The dental pulp was extracted by the fracture of the molar under sterile conditions and subsequently digested with an enzymatic solution composed of a 3 mg/mL collagenase type I (Gibco) and 4 mg/mL of dispase (Sigma) in Hank’s balanced salt solution (HBSS, Gibco, Carlsbad, CA, USA). The enzymatic digestion solution was incubated at 37 °C for 1 h, after which it was neutralized by the addition of Dulbecco’s Modified Eagle Medium (DMEM, Lonza, Basel, Switzerland) supplemented with 10% fetal bovine serum (FBS, HyClone, Logan, MA, USA). Immediately after that, cells were centrifuged at 430× *g* for 5 min, and the pellet was mechanically disrupted by carefully passing it several times through 18G needles (BD Microlance, Fisher Scientific, Hampton, VA, USA). After the disassociation step, the cells were seeded in 25 cm^2^ flasks (T25, Sarstedt, Nümbrecht, Germany) with DMEM supplemented with 10% fetal bovine serum (Gibco), 1% penicillin–streptomycin (Gibco), and 1% L-glutamine (Sigma) and cultured at 37 °C in 5% CO_2_. After reaching 80% confluence, cells were sub-cultured to 75 cm^2^ flasks (T75, Sarstedt). For the biocompatibility experiments, passages between 4 and 10 were used.

### 2.5. Fluorescent Labelling of Cells

In order to assess the effect of the functionalized PVDF surfaces on hDPSCs, 24 h after seeding, we stained the cells with labels specific for the actin cytoskeleton and nuclei. Briefly, specimens were washed with PBS and fixed in 4% formaldehyde (Thermo Scientific, 28908) for 10 min at room temperature. Next, we washed the samples twice with PBS before cell staining. Nuclei and F-actin were stained using Hoechst 33342 (NucBlue, Invitrogen, Waltham, MA, USA) and phalloidin (ActinRed 555, R37112, Thermo Scientific), respectively, following the manufacturer’s instructions. Briefly, samples were incubated for 1 h in a PBS solution containing 2 drops of NucBlue and ActinRed before washing them three times in PBS. Stained samples were kept in darkness until imaging using a Nikon TiU microscope (Tokyo, Japan). Acquired images were postprocessed using ImageJ [31].

### 2.6. Statistical Analysis

All experiments were performed at least 3 times (*n* = 3). Statistical analysis was performed using the IBM SPSS Statistical software (v. 26.0). The data from the different experimental conditions were compared using ANOVA followed by the Bonferroni or Games–Howell test depending on the homogeneity of the variances. The confidence intervals were set at 95% (*p* < 0.05), 99% (*p* < 0.01), and 99.9% (*p* < 0.001). The histograms show the average value and the standard deviation (SD) of each experimental sample. In turn, in the box plot figures, the centre line denotes the average value (50th percentile), showing the 25th to 75th percentiles of the dataset. The whiskers are set at Q1 − 1.5 IQR and Q3 + 1.5 IQR values. The data beyond these upper and lower bounds are considered outliers, marked with dots.

## 3. Results and Discussion

Among piezoelectric polymers, PVDF stands out due to its excellent mechanical properties, easy processing into different structures, chemical stability in physiological conditions, biocompatibility, and high piezoelectric response [8]. Depending on the preparation protocol used, this synthetic semicrystalline fluoropolymer can be obtained in five different phases, of which the β-phase is the one that displays the highest piezoelectric coefficient [34,35]. In addition, the application of an electric field to the PVDF films, a process named ‘poling’, further allows the formation of films with a net positive and a net negative side. Despite their great promise for the regeneration of damaged tissues, the synthetic nature and inherent hydrophobicity of these materials strongly limit their applicability for certain purposes, particularly those involving cell attachment. In addition, the variability in the protein adhesion to the PVDF surfaces with different polarities and surface charges [13], makes it difficult to unequivocally determine the impact of the surface potential on cells [36,37,38].

**Figure 2:**
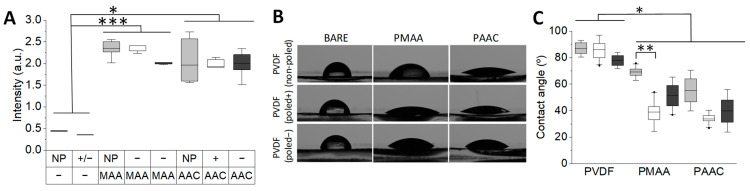
Physicochemical characterization of the PVDF-PMAA and PVDF-PAAC surfaces: (**A**) Surface functionalization with both acids is estimated from the absorption at 207 nm and 193 nm for PMAA and PAAC, respectively. “NP” stands for PVDF (non-poled), “+/–” for poled PVDF, “+” for positively poled PVDF, and “–” for negatively poled PVDF. (**B**) Images acquired for contact angle measurements and the corresponding quantification (**C**). In (**A**,**C**), grey corresponds to PVDF (non-poled), white to PVDF (poled+), and black to PVDF (poled−). Statistical significances are denoted with * for *p* ≤ 0.05, ** for *p* ≤ 0.01 and *** for *p* ≤ 0.001.

In order to create a biologically relevant environment and obtain PVDF surfaces with a coating that promotes cell adhesion, in the present work, we have established a new method for the chemical functionalization of this polymer. The previous literature describes approaches for the covalent binding of extracellular matrix proteins to the surface of PVDF [17]; however, these works neglect the polarity of the polymeric surfaces. With this in mind and given the importance of PVDF poling for its use in a biological context [5,13], our aim was to establish a protocol that yields equal densities of immobilized extracellular matrix proteins, independently of the PVDF poling state. The protocol is based on an oxidation step followed by the polymerization of either methacrylic (MAA) or acrylic (AAC) acid monomers [20], which results in the formation of surface-bound poly-MAA (PMAA) or poly-AAC (PAAC) chains. Firstly, PVDF surfaces were treated with UV light [16] in order to generate reactive hydroxyl groups and subsequently immersed in AAC or MAA solutions with acid concentrations tuned to obtain similar acid densities, independently of the PVDF polarity used. After optimization, approximately 500 per cm^2^ 10% (*v/v*) MAA solution for PVDF (poled+) and (poled-), and 8% (*v/v*) MAA for PVDF (non-poled), respectively, and 10% AAC for all PVDF variants were used (Supplementary Figure S1). After functionalization, we observed an increase in the absorbance at 207 nm for the PVDF-PMAA membranes and at 193 nm for the PVDF-PAAC membranes (Figure 2A), indicating the chemical modification of the surfaces. In turn, contact angle measurements revealed similar hydrophobicity of all non-modified PVDF surfaces (Figure 2B). After acid immobilization, the contact angle was significantly reduced in all cases, except for PMAA-functionalized PVDF (non-poled), which remained significantly hydrophobic (Figure 2C). Overall, it has been previously reported that such moderate hydrophilicity improves cell adhesion and biocompatibility [39].

**Figure 3:**
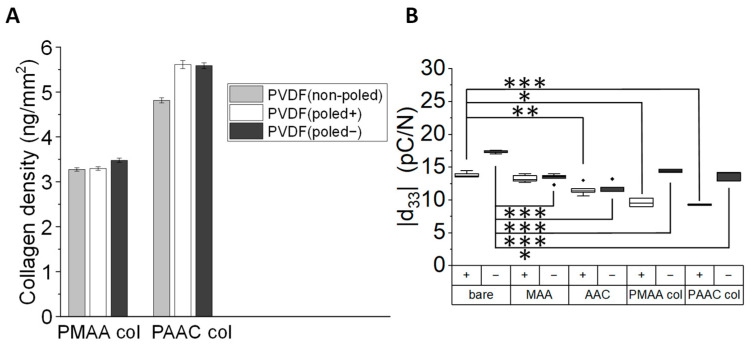
Quantification of the collagen immobilized on the PVDF surfaces and estimation of the piezoelectric properties: (**A**) Quantification of the amount of immobilized collagen type I on the functionalized PVDF substrates is measured by the BCA assay and reveals higher protein densities on the PVDF-PMAA than on the PVDF-PAAC surfaces. Nevertheless, similar protein densities are detected across the surfaces with different electric potentials. (**B**) Although there is a slight decay in the modulus of the piezoelectric coefficient (|d_33_|) of the functionalized PVDF films after the biofunctionalization process, particularly in case of PVDF (poled−), the resulting materials retain their piezoelectric properties to a great extent. Statistical significances are denoted with * for *p* ≤ 0.05, ** for *p* ≤ 0.01 and *** for *p* ≤ 0.001.

Next, we used the zero-length cross-linker 1-ethyl-3-(3-dimethylaminopropyl) carbodiimide (EDC) in combination with N-hydroxysuccinimide (NHS) to chemically activate the carboxylic groups exposed in the surface-bound PMAA and PAAC [28,29]. This carboxylic activation forms an anhydride intermediate in the case of the PMAA and an NHS-ester intermediate for the PAAC [40,41,42,43]. Next, we tested the ability of the system to covalently bind collagen type I. To do so, functionalized PVDF-PAAC and PVDF-PMAA surfaces were incubated with a 300 µg/mL collagen type I solution for 24 h. After washing the membranes to remove any unbound collagen, the immobilized amount was quantified using the bicinchoninic acid (BCA) assay (Figure 3A). The result revealed that all samples displayed an equivalent amount of immobilized protein, independently of their surface potential. Nevertheless, we detected slightly higher densities of collagen in the PVDF samples functionalized with AAC compared to those with MAA (Figure 3A). Although the exact cause of this discrepancy remains unknown, we hypothesize that it may be due to the different EDC/NHS activation mechanisms that have been described between PAA and PMAA brushes (Figure 1) [43].

Since piezoelectricity is central to tissue homeostasis and regeneration, and therefore one of the most attractive properties of PVDF for tissue engineering applications, we quantified the piezoelectric coefficient of the materials before and after each functionalization step. Our results reveal a statistically significant drop in the piezoelectric properties of PVDF (poled−) after the initial chemical modification (Figure 3B). In turn, the piezoelectric response of the negatively poled side (PVDF (poled+)) remained unaltered. After the immobilization of collagen molecules, the piezoelectric coefficient modulus (|d_33_|) of both surfaces, PVDF (poled−) and (poled+), remained similar, indicating that the materials retained their electroactive properties to a great extent. In addition, the piezoelectric coefficients of the functionalized PVDF films are in the same range as those of bone, which is the tissue of the body with the highest piezoelectric response [44].

To determine if the reduction in the piezoelectric response was caused by a decrease in the β-phase content of the films, we acquired FTIR-ATR spectra of the materials before and after the functionalization (Supplementary Figure S3A–C). Estimation of the relative β-phase content revealed minimal differences in the β-phase content of PVDF (poled−) before and after the functionalization, which remained around 60%. On the other hand, in PVDF (non-poled), the content was reduced from 56% to around 47% as a result of PMAA and PAAC functionalization, and, in the case of PVDF (poled+), the bare films contained 62% β-phase, which was reduced to 50% and 45% as a result of PMAA and PAAC functionalization, respectively (Supplementary Figure S3D).

**Figure 4:**
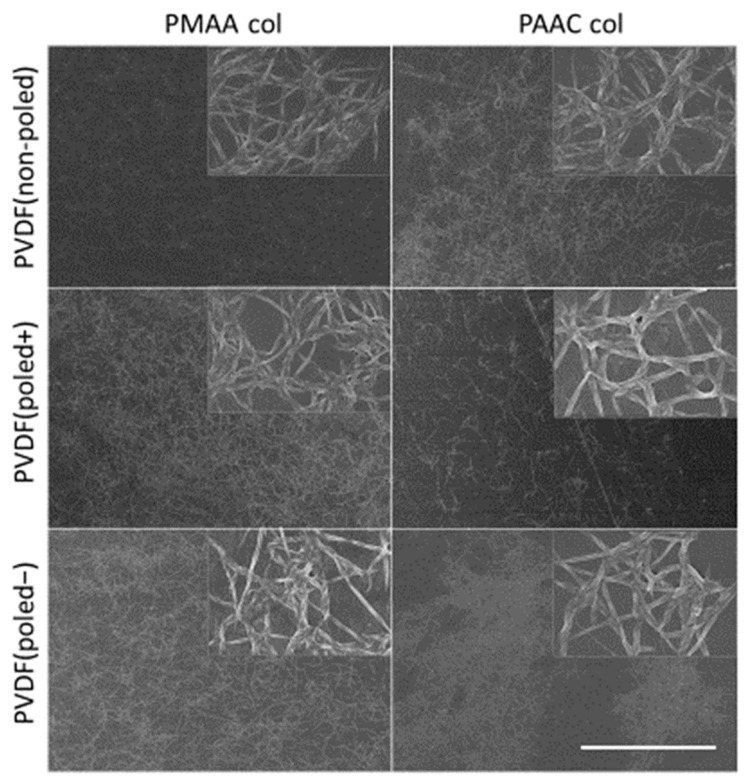
Scanning electron microscopy images of the samples with immobilized collagen type I. Collagen fibres appeared immobilized on the PVDF membranes functionalized with PMAA (left column) and PAAC (right column), forming a homogenous network across the surface, independently of its polarity. Scale bar represents 50 µm. Insets represent a 10-fold magnification.

Next, the samples were examined under the scanning electron microscope (SEM) to determine the distribution and organization of the immobilized collagen interface. The analysis revealed a homogenous collagen coating of the material with abundant fibrils attached to its surface (Figure 4), which were absent in the functionalized PVDF membranes without collagen incubation (Supplementary Figure S4). No further topographic differences were observed between the functionalized and non-functionalized surfaces. Nevertheless, the physicochemical analysis carried out cannot totally exclude the occurrence of other chemical modifications.

**Figure 5:**
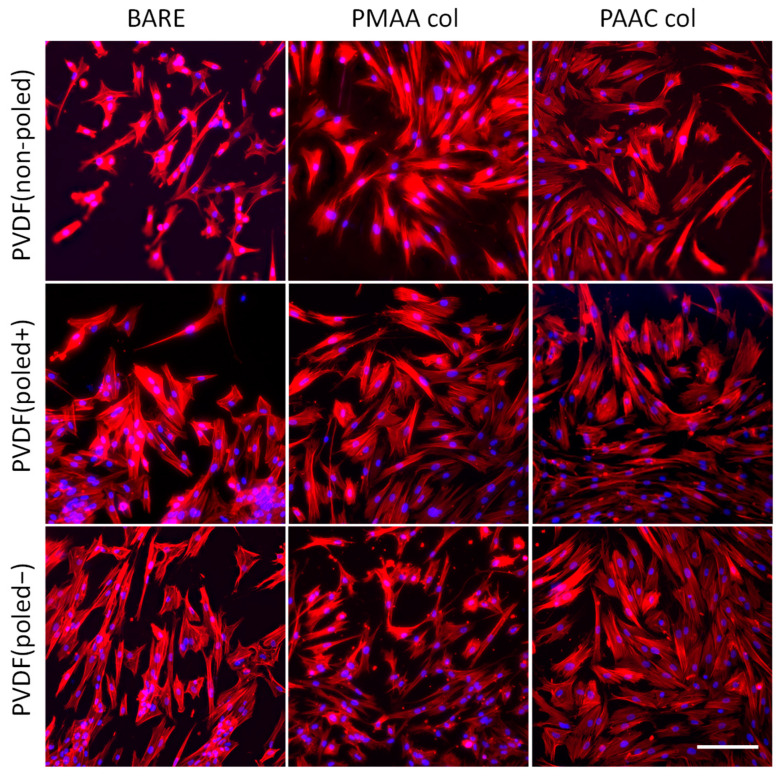
Fluorescently stained primary hDPSCs adhering to bare (left column) and functionalized PVDF surfaces (middle and right columns show PMAA-functionalized and AAC functionalized surfaces, respectively). hDPSCs are stained with Hoechst33342 (nuclei in blue) and phalloidin (filamentous actin in red). The morphological features of the cells reveal the good biocompatibility of the collagen-functionalized PVDF surfaces. Scale bar represents 150 µm.

Next, we tested the biocompatibility of the functionalized materials using human dental pulp stem cells (hDPSCs), a cell type of mesenchymal origin that has the ability to differentiate into a wide range of cell types [45]. Primary hDPSCs were seeded onto the collagen-coated substrates, and, 24 h later, samples were chemically fixed using formaldehyde, fluorescently stained and imaged under a widefield fluorescence microscope. The cell nuclei and actin cytoskeleton were labelled with Hoechst33342 and phalloidin, respectively. On all materials, independently of their poling state and chemical modification method (PAAC or PMAA), hDPSCs displayed morphologies characteristic of these cells, with a well-defined actin cytoskeleton and evenly distributed nuclei, suggesting healthy cellular architecture and behaviour (Figure 5) [28,40,41,42]. Interestingly, the footprint of the hDPSCs adhering to non-poled PVDF was larger when the surface was functionalized compared with the bare conditions (Figure 5, upper row). This is likely caused by the previously reported reduced collagen binding on non-poled PVDF, which likely limits the formation of focal adhesions and, consequently, cell spreading [13].

## 4. Conclusions

The described covalent immobilization of collagen type I on β-phase PVDF membranes creates a biomimetic composite that emulates bone in two key aspects of this tissue: it replicates the electrical characteristics of natural bone through the piezoelectric properties of PVDF while simultaneously presenting a biologically favourable surface that promotes cellular attachment. This modification addresses previous limitations associated with this polymer for biomedical applications, expanding both its functionality and biological compatibility and opening new possibilities for advanced tissue engineering approaches. In addition, this approach allows the piezoelectric stimulation of cells attached to otherwise identical surfaces, thereby facilitating the analysis of the transduction mechanisms of these types of signals.

## Figures and Tables

**Table 1:** Roughness and surface potential of the PVDF films used in the present study. Data were obtained from references [13,30].

	*Roughness*	*Net Surface Potential*
*PVDF (non-poled)*	18.23 ± 4.0 nm	0 V
*PVDF (poled+)*	17.74 ± 1.89 nm	6 V
*PVDF (poled−)*	15.98 ± 2.01 nm	−4 V

## Data Availability

Data will be made available upon request.

## Supplementary Materials

The following supporting information can be downloaded at https://www.mdpi.com/article/10.3390/biomimetics10020126/s1, Figure S1: Optimization of the methacrylic acid concentration; Figure S2: Calibration curve for the BCA assay; Figure S3: FTIR-based estimation of the relative β-phase content in bare and chemically-modified PVDF films; Figure S4: Scanning electron microscopy images of PVDF membranes.
