# Supplementary material for: Expanding the Applicability of Electroactive Polymers for Tissue Engineering Through Surface Biofunctionalization
Source: Biomimetics (Basel). 2025 Feb 19;10(2):126. doi: 10.3390/biomimetics10020126 (PMC11852601; doi:10.3390/biomimetics10020126)
Supplement: Supplementary file 1 [file biomimetics-10-00126-s001.zip › biomimetics-3395735-supplementary.docx]

**SUPPLEMENTARY INFORMATION**


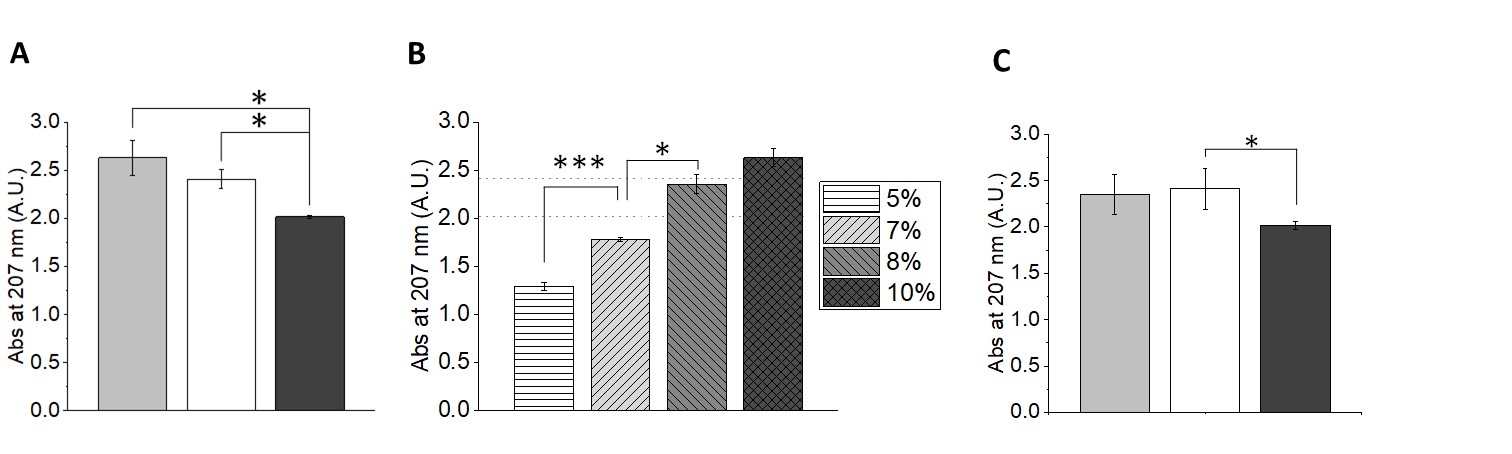


**Supplementary figure 1**. Optimization of the methacrylic acid (MAA) concentration used for the surface functionalization. A) A concentration of 10% MAA (v/v) resulted in higher PMAA concentration on the PVDF (non-poled) than on the positively and negatively poled PVDF substrates. B) Assays with 5, 7, 8 and 10% MAA were performed to determine the optimal acid concentration. C) The final functionalization was carried out with PMAA 8% and resulted in a slightly lower amount of PMAA in the negatively poled PVDF surfaces. In A) and C) grey represents PVDF (non-poled), white represents PVDF (poled +) and black represents PVDF (poled-). * for p ≤ 0.05 and *** for p ≤ 0.001.


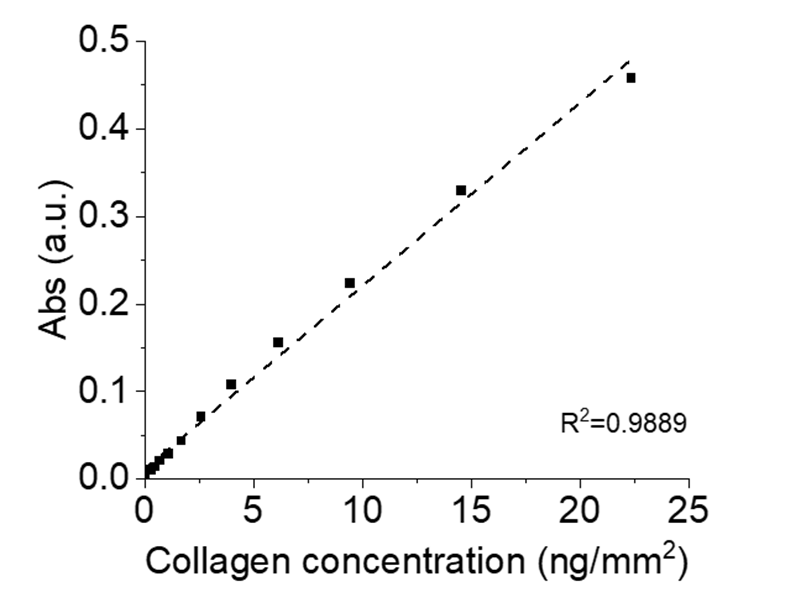


**Supplementary figure 2**. Calibration curve for the BCA assay made with native collagen type I.


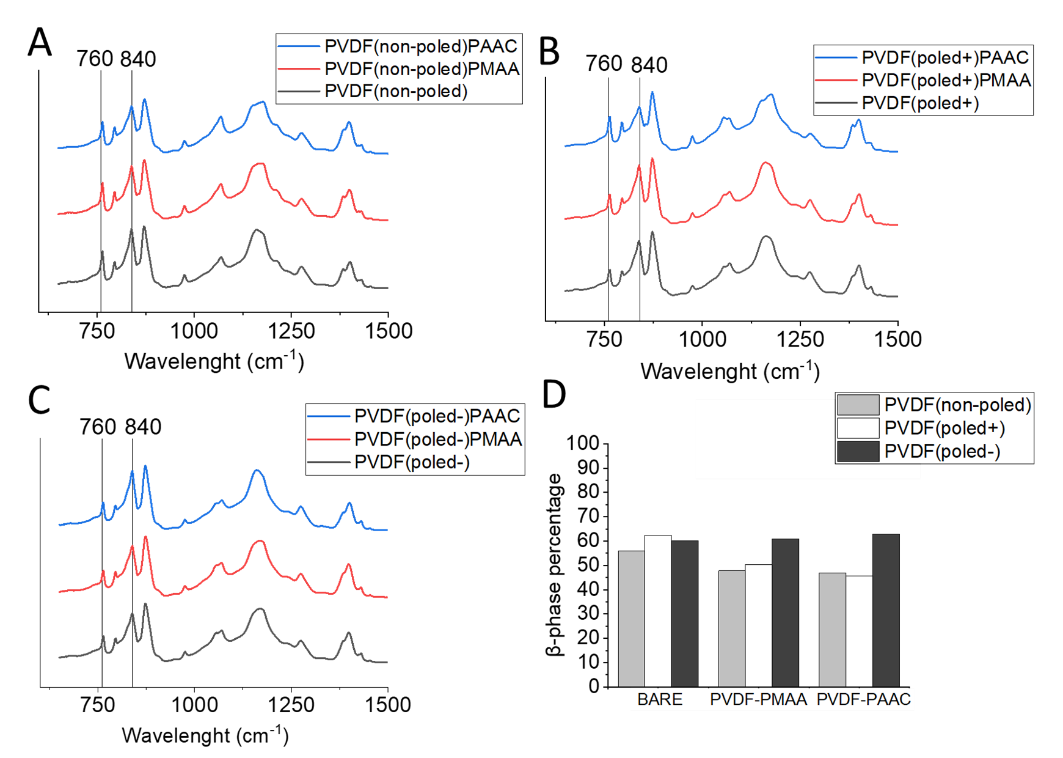


**Supplementary figure 3.** FTIR-based estimation of the relative β-phase content in bare and chemically-modified PVDF films. A) Spectra corresponding to PVDF (non-poled), B) PVDF (poled+) and C) PVDF (poled-). D) β-phase content estimation.

**Supplementary figure 4.** Scanning electron microscopy images of PVDF membranes functionalized with either PMAA (left column) or PAAC (right column) without collagen incubation. Scale bar represents 5 µm.
